# PRESIDENTIAL SYMPOSIUM: OPTIMIZING SURGICAL CARE FOR ALL OLDER ADULTS

**DOI:** 10.1093/geroni/igz038.1484

**Published:** 2019-11-08

**Authors:** Thomas Robinson, Ronnie Rosenthal

**Affiliations:** 1 University of Colorado, Aurora, Colorado, United States; 2 Yale School of Medicine, New Haven, Connecticut, United States

## Abstract

Our program will provide a detailed overview with an emphasis on the research aspects of the new Coalition for Quality in Geriatric Surgery, a project supported by the American College of Surgeons and the John A. Hartford Foundation. This project is a national endeavor which aims to systematically improve the surgical care of older adults by establishing a verifiable quality improvement program with standards based on best evidence focused on what matters most to the individual patient. We believe there is a critical need for safe, high-quality, patient-centered surgical care for older adults. Aging surgical patients have unique physiological needs, social needs and unique goals of care. We formed the Coalition to help hospitals meet these rising needs by setting and verifying interdisciplinary standards and developing outcome measures that matter to older patients, families and caregivers. In collaboration with our 50+ stakeholder organizations, we have set the standards, developed measures that matter, educated providers and patients, and created awareness about the surgical needs of older adults at all hospitals through the program. The geriatric surgery program, set to launch in the Summer of 2019, will use the four principles of continuous quality improvement: set standards, define the right infrastructure, collect rigorous data, and verify. The program not only improves perioperative care, but also impacts the full cycle of care for older adults. Our group has harnessed the power of networks through partnership and collaboration of all disciplines involved in the peri-operative care of older adults.

